# Evaluation of the Effects of Airborne Particulate Matter on Bone Marrow-Mesenchymal Stem Cells (BM-MSCs): Cellular, Molecular and Systems Biological Approaches

**DOI:** 10.3390/ijerph14040440

**Published:** 2017-04-20

**Authors:** Muhammad Abu-Elmagd, Mansour A. Alghamdi, Magdy Shamy, Mamdouh I. Khoder, Max Costa, Mourad Assidi, Roaa Kadam, Haneen Alsehli, Mamdooh Gari, Peter Natesan Pushparaj, Gauthaman Kalamegam, Mohammed H. Al-Qahtani

**Affiliations:** 1 Center of Excellence in Genomic Medicine Research, King Abdulaziz University, P.O. Box 80216, Jeddah 21589, Saudi Arabia; mourad.assidi@gmail.com (M.A.); rkadem@kau.edu.sa (R.K.); halsehli@kau.edu.sa (H.A.); mgari@kau.edu.sa (M.G.); mhalqahtani@kau.edu.sa (M.H.A.-Q.); 2 Department of Environmental Sciences, Faculty of Meteorology, Environment and Arid Land Agriculture, King Abdulaziz University, P.O. Box 80208, Jeddah 21589, Saudi Arabia; mans99@gmail.com (M.A.A.); mshamy@kau.edu.sa (M.S.); khoder_55@yahoo.com (M.I.K.); 3 New York University School of Medicine, Nelson Institute of Environmental Medicine, New York, NY 10987, USA; max.costa@nyumc.org

**Keywords:** particulate matter, BM-MSCs, cell proliferation, cell death, qRT-PCR, IPA

## Abstract

Particulate matter (PM) contains heavy metals that affect various cellular functions and gene expression associated with a range of acute and chronic diseases in humans. However, the specific effects they exert on the stem cells remain unclear. Here, we report the effects of PM collected from the city of Jeddah on proliferation, cell death, related gene expression and systems of biological analysis in bone marrow mesenchymal stem cells (BM-MSCs), with the aim of understanding the underlying mechanisms. PM_2.5_ and PM_10_ were tested in vitro at various concentrations (15 to 300 µg/mL) and durations (24 to 72 h). PMs induced cellular stress including membrane damage, shrinkage and death. Lower concentrations of PM_2.5_ increased proliferation of BM-MSCs, while higher concentrations served to decrease it. PM_10_ decreased BM-MSCs proliferation in a concentration-dependent manner. The X-ray fluorescence spectrometric analysis showed that PM contains high levels of heavy metals. Ingenuity Pathway Analysis (IPA) and hierarchical clustering analyses demonstrated that heavy metals were associated with signaling pathways involving cell stress/death, cancer and chronic diseases. qRT-PCR results showed differential expression of the apoptosis genes (BCL2, BAX); inflammation associated genes (TNF-α and IL-6) and the cell cycle regulation gene (p53). We conclude that PM causes inflammation and cell death, and thereby predisposes to chronic debilitating diseases.

## 1. Introduction

Air pollution is a major environmental risk factor that plagues both developing and developed nations alike. Toxic air pollutants can arise due to natural causes such as giant volcanic emissions, forest fires, gas emanating from radioactive rocks, or man-made, *viz*., industrial, chemical or traffic, emissions. Irrespective of the cause, toxic air pollutants adversely affect the health of an individual leading to morbidity and mortality [1]. The World Health Organization (WHO) reports that three million premature deaths occurred worldwide in 2012, due to air pollution. Rising levels of urban air pollution in South-East Asia and Eastern Mediterranean countries have become a matter of great concern [2]. Figures from WHO identify the Kingdom of Saudi Arabia as the country with the highest annual mean concentration of fine particulate matter from urban areas in 2014, with a concentration of 127 µg/m^3^ [3].

Visible smoke or dust can be avoided to some extent, but it is practically impossible to achieve daily protection from exposure to the invisible suspended particulate matter (PM). PMs with size ranges from ultrafine (D_p_ < 0.1 µm) to coarse (100 µm in diameter) [4], induce different health hazards. PM with an aerodynamic diameter equal to or less than 10 µm in size (PM_10_) can access the human body with ease and proceed to affect different organ systems [5]. Coarse particles (2.5–10 µm) deposit in the nasopharyngeal region and the upper regions of the lung; while fine (<2.5 µm) and ultrafine (<1 µm) particulates may penetrate deep into the alveolar region [6]. Dermatological (irritations, rashes, pigmentations) [7], ocular (corneal irritation, conjunctivitis) [8], respiratory (cough, allergic rhinitis, chronic obstruction of the airways) [9,10,11], cardiovascular (palpitations, atherosclerosis) [12,13], neurological (cerebrovascular accidents) [14,15,16,17,18], immunological (allergy, inflammation) [19,20], metabolic (*Diabetes mellitus*, dyslipidaemia) diseases [21,22] and cancers [23,24] are all associated with PMs.

At the cellular level, various particulate sizes including PM_2.5_ and PM_10_ can induce cytotoxic effects including DNA oxidative damage and stimulation of pro-inflammatory factors [2]. Exposure to PM_10_ in bronchial epithelial cells led to the activation of cholesterol and lipid synthesis [25]. PMs are also reported to cross the fetomaternal interface leading to developmental delay and/or malformations [2,26]. Stem cells play an active role not only during the embryonic development but also in later life where they are associated with continuous turnover of cells of the skin, haematopoietic tissue and the intestinal system [27]. In addition, adult stem cells continue to reside in special niches within various tissues and contribute to repair when required. Since stem cells have prolonged survival capacity, they are vulnerable to the long-standing toxic effects of biological, chemical or environmental agents. The direct effects of PM on stem cells remains hitherto unexplored. As such, in the present study, we aimed to evaluate the effects of the PM_2.5_ and PM_10_ collected from the city of Jeddah on bone marrow-derived mesenchymal stem cells (BM-MSCs) in vitro in relation to their proliferation and cell death. Furthermore, the predictive effects of PM on the various signaling pathways were analyzed using the Ingenuity Pathway Analysis (IPA).

## 2. Materials and Methods

### 2.1. Particle Sample Collection

Dust samples were collected from the King Abdulaziz University (KAU) campus (south of the city of Jeddah, Saudi Arabia). Metal composition analysis was carried out according to our earlier published report [25]. Briefly, air particles of PM_2.5_ and PM_10_ were collected on 5300 polypropylene filters using high volume air sampler (The Staplex Company, Staplex Air Sampler Division, Brooklyn, NY, USA), with an inlet for PM_2.5_ and PM_10_ at a constant flow rate of 900 I/min and these were used for in vitro studies.

### 2.2. Element Metal Analysis

In a temperature- and humidity-controlled weighing room, the mass on the Teflon filters was determined using a microbalance (model MT5, Mettler-Toledo Inc., Hightstown, NJ, USA). Analysis of the metal concentrations was carried out as described by Maciejczyk and Chen, 2005 [28]. Briefly, a non-destructive X-ray fluorescers (XRF) spectrometer (EX-6600-AF, Jordan Valley, Austin, TX, USA) with five secondary fluorescers (Fe, Ge, Mo, Si, and Ti) and spectral software XRF2000 V3.1 (U.S. EPA and ManTech Environmental Technology, Inc., Research Triangle Park, NY, USA) were used to determine the metal element concentrations.

### 2.3. Particle Extraction

A modified aqueous method reported by Duvall et al., 2008 [29] was followed to extract the PM_2.5_ and PM_10_. The dust was extracted from the polypropylene filters and each filter was dampened with 25 mL of 70% EtOH after which they were sonicated in 100 mL of distilled H_2_O for two hours. The particles were dried by lyophilization, weighed and stored at −80 °C. The diameter for the collected dust used for the in vitro cellular exposure for PM_10_ ranged from 2.5 µm to 10 µm and <2.5 µm for the PM_2.5_.

### 2.4. Culture of Bone Marrow Mesenchymal Stem Cells (BM-MSCs)

Bone marrow aspirates were obtained from 5 male patients aged between 52 to 64 years, who were earlier treated for osteoarthritis and were attending surgical procedures at the Department of Orthopaedics, KAU Hospital, Jeddah, Saudi Arabia. The samples were collected following Institutional Ethical Committee approval (11-557/KAU) and informed patient consent. In-house-derived primary cultures of BM-MSCs that were characterized earlier were used in the present study. BM-MSCs were maintained in culture using Dulbecco’s modified Eagle’s medium (Sigma, St. Louis, MO, USA), supplemented with 10% fetal bovine serum [30], 2 mM Gluta-Max (Life Technologies, Carlsbad, CA, USA) and antibiotic solution (Penicillin, 100 U/mL; Streptomycin 100 µg/mL—Sigma) under standard culture conditions of 37 °C and 5% Carbon Dioxide (CO_2_) in atmospheric air. Basic fibroblast growth factor (bFGF; Peprotech, UK) at 5 ng/mL was added to culture medium to facilitate BM-MSCs expansion.

### 2.5. Cell Morphology

BM-MSCs from early passages (P3-P4) were seeded at a density of 2 × 10^4^ cells/well in 24-well tissue culture plates and incubated in a complete culture medium under standard culture conditions. Measured quantities of both PM_10_ and PM_2.5_ were mixed with known volumes of cell culture media and sonicated for 5 min which served as the main stock solutions of PM. Sub-dilutions for the required concentrations were prepared in fresh culture media. Briefly, PM_10_ or PM_2.5_ were added to the wells at different concentrations of 15, 25, 50, 150, 300 µg/mL and cultured for 24 h, 48 h and 72 h under standard culture conditions as described above. The cells were observed regularly and any changes in cell morphology were imaged using inverted phase contrast optics (Nikon Instruments, Tokyo, Japan).

### 2.6. Cell Proliferation

BM-MSCs from early passages (P3–P4) were seeded at a density of 2 × 10^4^ cells/well in 24-well tissue culture plates and cultured as above. The cells were exposed to similar concentrations of PM_10_ or PM_2.5_ and time durations as above. At the end of the respective time points, changes in cell proliferation—if any—were analyzed using 3-(4,5-dimethylthiazolyl-2)-2,5-diphenyltetrazoliumbromide assay (MTT; Sigma). Briefly, 10 µL of MTT reagent was added to 100 µL of the freshly-changed medium and incubated for four hours. The tetrazolium salt is reduced due to the action of the mitochondrial dehydrogenase to form water-insoluble formazan crystals, which were dissolved using the solvent, and absorbance at 570 nm was obtained using spectrophotometry (SpectraMax^®^ i3x, Molecular Devices, Sunnyvale, CA, USA).

### 2.7. Ingenuity Pathway Analyses (IPA) of Heavy Metals in the Dust Particles

Dust particles collected from city of Jeddah have been reported to contain heavy metals, namely Chromium (Cr), Manganese [31], Cobalt (Co), Arsenic (As), Lead [16], Cadmium (Cd), Nickel (Ni), and Strontium (Sr) [32]. The Ingenuity Pathway Analysis (IPA) Knowledgebase (Qiagen, Redwood City, CA, USA) was used to decipher the influence of these heavy metals on specific genes regulated in BM-MSCs. The gene list obtained for each heavy metal was then subjected to core analyses in IPA and the results were further clarified using Fisher Exact Test (*p* < 0.05) to obtain the involved pathways and networks. Additionally, results from the core analysis were compared using the Comparison Analyses Module in IPA, and these results were further clarified using Benjamini-Hochberg Correction (*p* < 0.05) to identify cellular and disease-specific pathways. Heatmaps and hierarchical clustering (complete linkage) were then generated based on the log2 transformed ratios, using Genesis Software (Release 1.7.7, Institute for Genomics and Bioinformatics, Graz University of Technology, Graz, Austria) [33].

### 2.8. Quantitative Real-Time Gene Expression Analysis (qRT-PCR)

Total RNA was extracted from controls and treated samples (PM_2.5_ µm and PM_10_ µm; at 150 µg/mL concentration for 48 h) using RNeasy mini kit (Qiagen, Redwood City, CA, USA), in accordance with the manufacturer’s instructions, including on column treatment with DNase-I. Quantity and quality of the isolated RNA were assessed using Nanodrop (Nanodrop Technologies, Wilmington, DE, USA). First strand complementary DNA (cDNA) was synthesized with random hexamers using the reverse transcription system (Promega, Madison, WI, USA). Primers were obtained from previously-published work, and the primer sequences are given in Table 1. qRT-PCR was carried out with SYBR Green master-mix (Life Technologies, Carlsbad, CA, USA) using the ABI StepOnePlus™ (Applied Biosystems, Foster City, CA, USA). Relative quantitation was undertaken using the comparative 2 ^−ΔΔCt^ method.

### 2.9. Statistical Analysis

Statistical analysis was performed using statistical package for social sciences (SPSS, IBM Analytics, Armonk, NY, USA) version 21. The differences between the experimental and control groups were analyzed using one-way ANOVA. The results were expressed as a mean ± SEM (standard error of the mean) from a minimum of three experimental replicates. The asterisk (*) indicates statistical significance of *p* < 0.05.

## 3. Results

### 3.1. Cell Morphology

**Figure 1:**
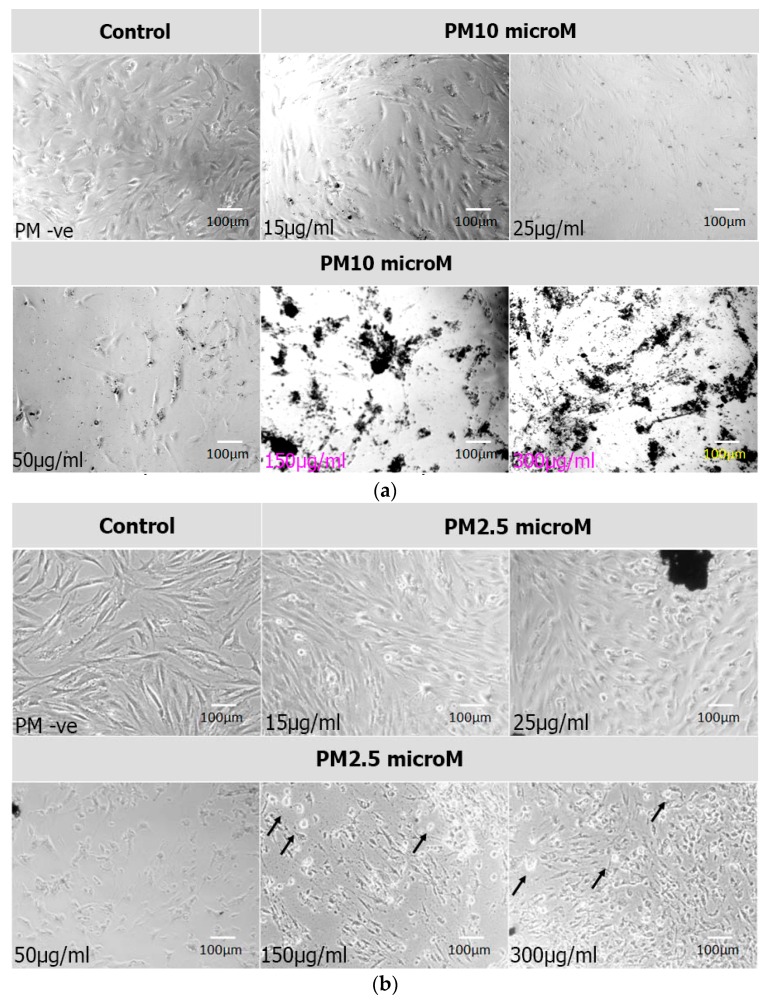
Cell morphology. Phase contrast images of bone marrow mesenchymal stem cells (BM-MSCs) either untreated [(control/negative of the airborne particulate matter (PM-ve)] or treated for 72 h with two different PM sizes [PM_10_ microM (**a**) and PM_2.5_ microM (**b**)] at different concentrations (15, 25, 50, 150 and 300 µg/mL), demonstrating the adherence of the PMs to the BM-MSCs which led to obscuring their complete morphology. This was more pronounced with PM_10_ (**a**) than PM_2.5_ exposure. The increase in PMs’ concentrations led to a decrease in cell numbers which was associated with morphological cellular changes in the treated BM-MSCs. These changes included cell shrinkage, thinning, and fragmentations which led to cell death (indicated in Figure 1b by arrows, bottom row, panels 2 and 3 in which dead cells appear rounded and translucent). The morphological changes were more evident with PM_2.5_ exposure (**b**), (Magnification 100×).

Phase contrast microscopy of the BM-MSCs exposed to PM_2.5_ and PM_10_ and cultured for 24 h, 48 h or 72 h showed a general increase in cell numbers with PM_2.5_, compared to the control. A general decrease in cell numbers was observed with PM_10_ compared to the controls. The PMs were tethered to the cell surface, and the cellular morphology was obscured, especially with PM_10_. Increase in the concentrations of PM and their exposure time led to a proportionate increase in cell death. This was more evident in PM_10_ compared to PM_2.5_. The cells showed features of stress including cell shrinkage and membrane disruptions leading to their death (Figure 1a,b).

### 3.2. Cell Proliferation (MTT Assay)

**Figure 2:**
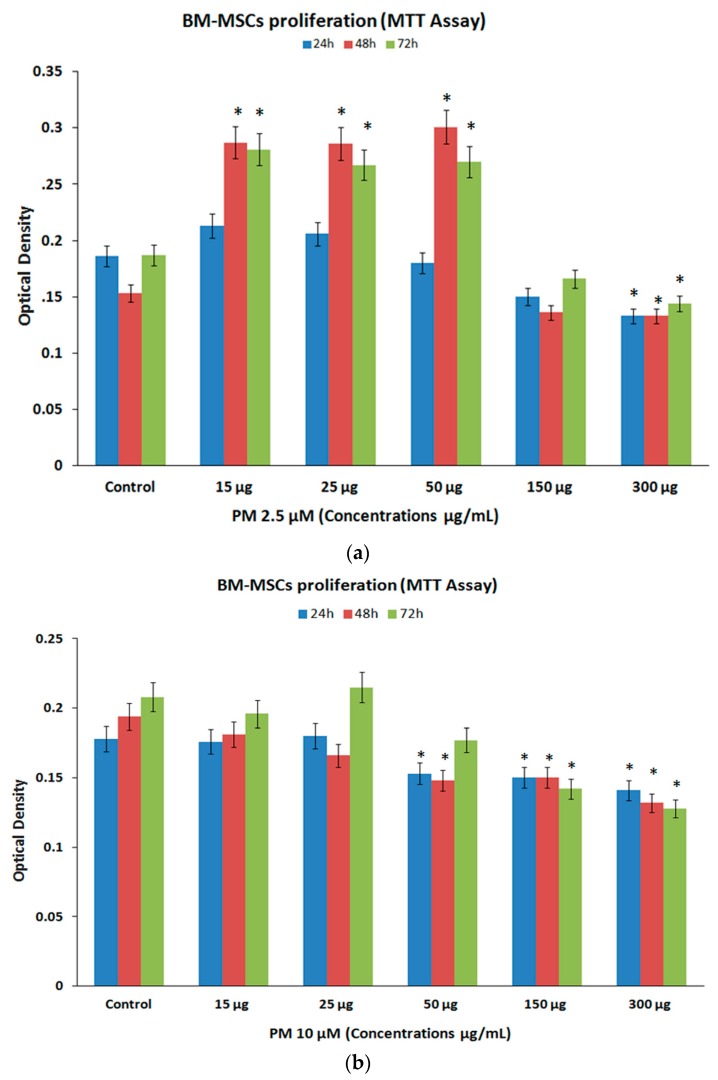
Cell proliferation/inhibition (MTT assay). BM-MSCs treated with PMs at different concentrations (15, 25, 50, 150 and 300 µg/mL) at 24 h, 48 h and 72 h showed increases in cell proliferation with PM_2.5_ (**a**), except for the highest concentration; while PM_10_ demonstrated inhibition of cell proliferation compared to the untreated controls (**b**). The values are expressed as mean ± SEM from triplicate samples of three independent experiments. The observed increases or decreases in cell proliferation with PMs were statistically significant. Asterisks “*” indicate statistical significance of *p* < 0.05.

The BM-MSCs exposed to PM_2.5_ demonstrated increases in cell proliferation at lower concentrations (15 µg/mL, 25 µg/mL, 50 µg/mL), and moderate decreases in proliferation at higher concentrations (150 µg/mL, 300 µg/mL). The mean percentage increases for PM_2.5_ at 24 h, 48 h and 72 h were 14.52%, 87.58%, 50.27% for 15 µg/mL; 10.75%, 86.93%, 42.78% for 25 µg/mL; and 96.73%, 44.39% for 50 µg/mL respectively, all these increases at 48 h and 72 h were statistically significant (*p <* 0.05). The mean percentage decreases for PM_2.5_ at 24 h, 48 h and 72 h were 19.35%, 11.11%, 11.23% for 150 µg/mL and 28.49%, 13.07%, 22.99% for 300 µg/mL respectively, these decreases were all statistically significant (*p* < 0.05) (Figure 2a).

The BM-MSCs treated with PM_10_ demonstrated only decreases in cell numbers at higher concentrations compared to their untreated control. The mean percentage decreases for PM_10_ at 24 h, 48 h and 72 h were 14.04%, 23.71%, 14.90% for 50 µg/mL; 15.73%, 22.68%, 31.73% for 150 µg/mL and 20.79%, 31.96%, 38.46% for 300 µg/mL respectively. However, only the decreases observed at 24 h for the highest concentration (300 µg/mL) as well as the decreases observed at 48 h and 72 h for all three higher concentrations (50 µg/mL, 150 µg/mL, 300 µg/mL) were statistically significant (*p* < 0.05) (Figure 2b).

### 3.3. IPA Analysis for Associated Genes and Networks

**Figure 3:**
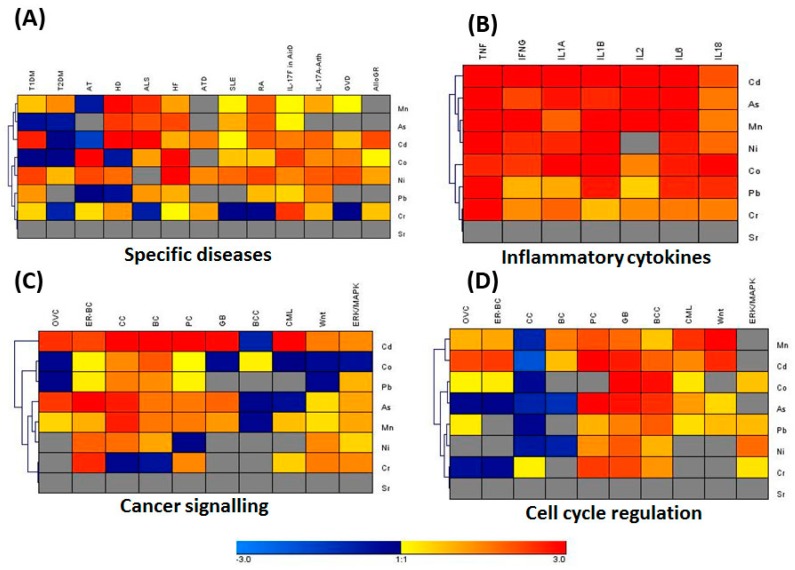
The ingenuity pathway analysis (IPA) of genes modulated by heavy metals. IPA knowledgebase was used to obtain genes that are differentially regulated by heavy metals present in the dust particles. Comparison analysis module of IPA was also utilized to compare the results obtained using the core analysis for each heavy metal (heavy metals list is on the right-hand side of each map in **A**–**D**). Hierarchical clustering was carried out based on the comparison analysis results using Genesis Software in specific diseases in panels (**A**) including Type I and Type II of *Diabetes mellitus* (TI-DM and TII-DM), rheumatoid arthritis (RA), Huntington’s disease (HD), amyotrophic lateral sclerosis (ALS), and systemic lupus erythematosus; (**B**) immune regulation (inflammatory cytokines); (**C**) cancer signaling; and (**D**) cell cycle regulation.

PM from the city of Jeddah was reported to be rich in a significant number of heavy metals [33]. Using the core IPA analysis, genes that are differentially regulated in BM-MSCs and other primary cells and tissues by heavy metals present in the dust particles were identified. Furthermore, the comparison analysis of the core IPA results showed that heavy metals present in dust particles differentially regulate various canonical pathways such as cancer signaling, cell cycle regulation, stress and injury signaling, inflammatory cytokine signaling and an array of disease-specific pathways (Figure 3A–D). The heatmap and complete hierarchical clustering (HCL) analyses showed that heavy metals, such as Cd, Pb, As, Mn, Ni, Cr, and Co upregulated genes associated with cancer signaling. The disease-specific genes in Type I and Type II of *Diabetes mellitus* (TI-DM and TII-DM), Rheumatoid Arthritis (RA), Huntington’s Disease (HD), Amyotrophic Lateral Sclerosis (ALS), and Systemic Lupus Erythematosus were differentially regulated by Cd, As, Ni, Co, and Mn (Figure 3A). Interestingly, our results suggested that, with the exception of Strontium (Sr), all the heavy metals upregulated the expression of proinflammatory cytokines such as TNF-α, IFN-γ, IL-1α, IL-1β, IL-2, IL-6 and IL-18 (Figure 3B). The genes related to ovarian cancer (OVC), colorectal cancer (CC), estrogen-dependent breast cancer (ER-BC), prostate cancer (PC), Glioblastoma (GB), chronic myeloid leukemia (CML), as well as Wnt and ERK/MAPK signaling pathways were potentially upregulated (Figure 3C). Furthermore, the genes responsible for the regulation of cell cycle, stress and injury were significantly upregulated by Cd, Pb, As, Cr, and Mn (Figure 3D).

### 3.4. Gene Expression Analysis of the Proinflammatory Markers (qRT-PCR)

**Figure 4:**
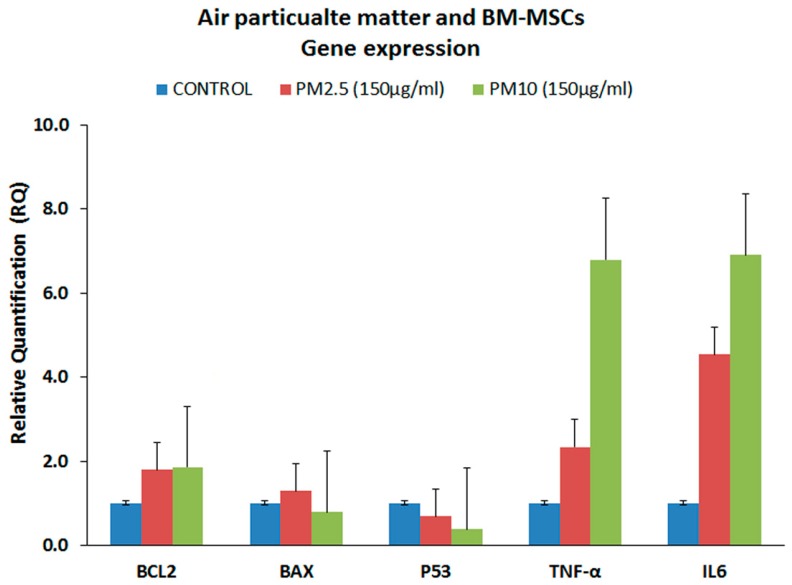
**Gene expression analysis.** qRT-PCR analysis of BM-MSCs showing the BCL2, BAX, p53, TNF-α and IL-6 gene expression profile following treatment with 150 µg/mL of PM_2.5_ and PM_10_ for 48 h. GAPDH was used as the internal control and the data quantified using the comparative 2^−ΔΔCt^ method. The values are expressed as mean ± SEM from triplicate samples of two independent experiments.

Gene expression analysis for some of the inflammation, cell death and cancer/cell cycle regulation related genes were undertaken using qRT-PCR. The inflammation related markers namely, TNF-α and IL-6 were upregulated compared to control (Figure 4). TNF-α was increased by 1.34 and 5.80-fold following treatment with PM_2.5_ and PM_10_ respectively. IL-6 was increased by 3.54 and 5.90-fold following treatment with PM_2.5_ and PM_10_ respectively. Gene expression that showed more than two-fold upregulation was statistically significant. The anti-apoptotic BCL2 gene showed mild upregulation, while the pro-apoptotic BAX was downregulated. In addition, the tumor suppressor gene, namely p53, which is involved in cell cycle regulation, was also downregulated compared to the control (Figure 4). However, the associated fold increases or decreases of these genes were not statistically significant.

## 4. Discussion

Airborne PMs, collected from the city of Jeddah in Saudi Arabia, contain high levels of Cr, Mn, Sr, Co, As, Pb, Cd, and Ni are implicated in many allergic, inflammatory, genetic and epigenetic disorders [22,34,35,36]. Mesenchymal stem cells are an invaluable and promising resource for use in regenerative medicine and thus understanding the mechanisms through which these cells regulate the immune response is vital. Interestingly, the mesenchymal stem cells have been reported to regulate the immune response for several human diseases through mediating several soluble factors and cell-cell contact [37,38,39,40,41]. Recently, some studies and clinical trials utilized the BM-MSCs to treat human diseases such as amyotrophic lateral sclerosis and Huntington’s disease [40,42]. In the current study, we evaluated the direct effects of these PMs on cellular functions of BM-MSCs, using two different sizes (PM_2.5_ and PM_10_). Our results revealed that several cellular morphological changes including cell shrinkage, thinning, and fragmentations (led to cellular death) were induced. An earlier in vitro study testing urban particles on alveolar macrophages reported their reversal to a more immunoreactive phenotype, and induction of apoptosis upon incubation for 24 h at 100 µg/mL and 200 µg/mL concentrations, respectively [22]. In addition, alveolar epithelial cell line (C10) exposed to higher concentrations (50 µg/cm^2^) of PM_2.5_ resulted in an increase in the sub-G0/G1 phases indicative of both apoptotic and necrotic cell death [43]. Increased expression of two death domain proteins namely, receptor interacting protein (RIP) kinases and Fas Associated protein with Death Domain (FADD) complex acting in concert with caspase-8 were implicated in the PM_2.5_ induced apoptosis [43]. Though no direct apoptotic assays were carried out in this study, the morphological characteristics indicated that the cell death could be attributed in part to apoptosis. However, in the present study the TNF-α gene expression was increased with both PM_2.5_ and PM_10_, and interestingly, RIP is reported to interact with either TNF receptor signalling or FADD to induce apoptosis [44].

BM-MSCs proliferation increased upon treatment with lower concentrations of PM_2.5_ in this study. Alveolar epithelial cells exposed to 10 µg/cm^2^ of PM_2.5_ for 24 h increased the cells in ‘S’ phase of the cell cycle which was attributed to injury/inflammation leading to compensatory proliferation [43]. Human alveolar basal adenocarcinoma cell line (A549) and human non-small lung carcinoma cell line (H1299) exposed to a conditioned medium of PM_2.5_ (50 μg/cm^2^ for 72 h) demonstrated an increase in proliferation [34]. Increased cell proliferation of these cancer cell lines was contributed to by interleukin 1-beta (IL1-β) mediated mitogen associated protein kinase (MAPK) signaling [34]. In contrast to the lower concentrations of PM_2.5_, higher concentrations of both PM_2.5_ and PM_10_ decreased BM-MSCs proliferation. Significant decrease in cell viability was reported with alveolar macrophages tested with urban particles in vitro at 200 µg/mL concentration for 24 h [22]. Similarly, the alveolar epithelial cells exposed to 50 µg/cm^2^ of PM_2.5_ for 24 h decreased the cells in the ‘S’ phase of the cell cycle [43]. Furthermore, the human colon cancer cell line HCT116 and the human embryonic kidney cell line HEK293T, exposed to PM_10_ at 400 µg/mL and 60 µg/mL concentrations, respectively, for 48 h, demonstrated 50% inhibition in their proliferation [32]. Results from this study and those cited above indicate that inhibition in cell proliferation is associated with higher concentrations of PM. However, it was noted that the decrease in cell proliferation in this study was not very high, particularly when considering the observed cellular changes. This could be explained by interference in the optical density/absorbance due to the PM that was tethered to the cells. Mostly, these decreases in cell proliferation were associated with cell death either due to apoptosis and/or necrosis. However, the expression of BCL2, BAX and p53 genes in our study did not support apoptotic patterns of cell death, indicating a role for other mediators of cell death. Although interaction with TNF receptor with RIP and FADD is reported to cause apoptosis [44], it is certainly necessary to undertake either an early time point of study for apoptosis or screen for additional pathways of cell death in subsequent studies.

Chronic exposure to airborne PMs can cause detrimental health effects, by decreasing normal cells including stem cells that offer general protection and promote cancer cell growth and migration even at smaller concentrations. Heavy metals, among others, are the most important toxic pollutants in the PM that affect many cellular functions and signaling pathways contributing to a disease. The systems biological analysis, using the IPA knowledgebase, helped us to decipher the disease-associated functions of heavy metals present in the PMs. The heavy metals have been found to trigger the production of proinflammatory cytokines such as TNF-α, IFN-γ, IL-1α, IL1-β, IL-2, IL-6 and IL-18 causing both acute and chronic inflammation. Furthermore, the heavy metals present in the PMs were shown to induce signaling pathways implicated in an array of cancers afflicting humans. More importantly, this inflammation is the basis for various diseases such as allergies, asthma, rheumatoid athritis, sytemic lupus erythematosus, Huntington’s disease, cardiovascular diseases, Alzheimer’s disease, *Diabetes mellitus* and a wide variety of cancers [45]. This was in tandem with our current gene expression analysis, which exhibited increased expression of inflammatory cytokines namely TNF-α and IL-6 following treatment of BM-MSCs with PM_2.5_ and PM_10_. These results are in concordance with a previously-reported study in which PM_2.5_ and PM_10_ increased the expression levels of the inflammatory markers including TNF-α and IL1-β in endothelial progenitor cells [46], IL-6 and IL-8 in monocytes [47], IL-6 in Kupffer cells [48] and TNF-α, IL-6 and IL-8 in lung epithelial cells [49]. The mechanism through which the airborne particulates induced apoptosis and increased the expression level of the inflammatory markers was attributed to triggering the formation of the reactive oxygen species (ROS) via impairment of P-Akt signaling [46,49,50]. Several sources of evidence also suggest that diseases are linked to ROS formation in response to air pollution [51]. It is possible, in our experiments, that a similar mechanism of ROS formation is attributed to inducing the pro-inflammatory markers TNF-α and IL-6 after treating the BM-MSCs with the PM. In osteocytes, which are mesenchymal in origin, Co-Cr-Mo alloy particles were able to induce pro-inflammatory markers including TNF-α [31].

The differences in cellular effects observed in the present study following exposure to PM_2.5_ and PM_10_ could be attributed to the particokinetic effects [52]. Use of equal mass concentrations (µg/mL) between different particles will exhibit tremendous differences by orders of magnitude in the number of particles or their surface area concentration and therefore the outcome of the cellular effects. Unlike chemicals where the physico-chemical property remains largely unchanged in a given solution, the particle solution dynamics are complex, given the differences in particle size, shape and surface chemistry. Therefore, incorporation of metrics such as diffusion, settling velocity and agglomeration of the particles as well as the fluid dynamics including media viscosity and density are essential in the determination of effective doses to study cellular effects.

## 5. Conclusions

Airborne PM in the city of Jeddah contains different heavy metals and environmental exposure can be detrimental to health. The present study provides an insight into the effects of PM on stem cells, that normally participate in the repair mechanisms against injury, inflammation and diseases. In vitro effects of two different sizes of PM (PM_2.5_ and PM_10_) indicated that lower concentrations increased, and higher concentrations decreased, BM-MSCs’ cell proliferation. The PM also increased pro-inflammatory cytokines, which are usually associated with various acute and chronic disease pathogenesis. The IPA analysis identified that PM augments various signaling pathways such as inflammation, cancer, cell cycle regulation and other set of diseases including *Diabetes mellitus*, rheumatoid arthritis, Huntington’s disease, amyotrophic lateral sclerosis, and systemic lupus erythematosus. Further investigation is needed to understand the mechanism of cell death observed in the present study and the predicted cell signaling pathways.

## Figures and Tables

**Table 1:** Primers’ sequence of genes tested by qRT-PCR including the house-keeping gene GAPDH as an internal control, anti-apoptotic BCL2, pro-apoptotic BAX, tumor suppressor p53 and the inflammation-related markers TNF-α and IL-6.

Gene	Primer Sequence
GAPDH	F: 5′-ACCACAGTCCATGCCATCAC-3′ R: 5′-TCCACCACCCTGTTGCTGTA-3′
BCL2	F: 5’-GGCTGGGATGCCTTTGTG-3’ R: 5’-CAGCCAGGAGAAATCAAACAGA-3’
BAX	F: 5’-TGGAGCTGCAGAGGATGATTG-3’ R: 5’-GCTGCCACTCGGAAAAAGAC-3’
p53	F: 5’-GCGCACAGAGGAAGAGAATC-3’ R: 5’-CTCTCGGAACATCTCGAAGC-3’
TNF-α	F: 5′-GGT-GCTTGT-TCC-TCA-GCC-TC-3′ R: 5′-CAG-GCA-GAAGAG-CGT-GGT-G-3′
IL-6	F: 5′-CCACTCACCTCTTCAGAA-3′ R: 5’-GCGCAAAATGAGATGAGT-3’

## Abbreviations

ALS	Amyotrophic Lateral Sclerosis
As	Arsenic
bFGF	Basic fibroblast growth factor
BM-MSC	Bone-marrow mesenchymal stem cells
CC	Colorectal cancer
Cd	Cadmium
cDNA	Complementary DNA
CML	Chronic myeloid leukemia
Co	Cobalt
CO_2_	Carbon Dioxide
Cr	Chromium
ER-BC	Estrogen-dependent breast cancer
FADD	Fas Associated protein with Death Domain
GB	Glioblastoma
HCL	Heatmap and complete hierarchical clustering
HD	Huntington’s Disease
IPA	Ingenuity Pathway Analysis
Mn	Manganese
Mo	Molybdenum
MTT	3-(4,5-dimethylthiazolyl-2)-2,5-diphenyltetrazoliumbromide assay
Ni	Nickel
NSCLC	Non-small-cell lung cancer cells
OVC	Ovarian cancer
ORS	oxygen reactive species
Pb	Lead
PC	Prostate cancer
PM	Particulate matter
qRT-PCR	Quantitative real-time gene expression analysis
RIP	Receptor interacting protein
ROS	Reactive oxygen species
RA	Rheumatoid Arthritis
SLE	Systemic lupus erythematosus
SPSS	Statistical package for social sciences
Sr	Strontium
TIDM	Type I Diabetes Mellitus
TIIDM	Type II Diabetes Mellitus
WHO	World Health Organization
XRF	X-ray Fluorescence

## References

[1] Kelly F.J., Fussell J.C. (2015). Air pollution and public health: Emerging hazards and improved understanding of risk. Environ. Geochem. Health.

[2] Valavanidis A., Fiotakis K., Vlachogianni T. (2008). Airborne particulate matter and human health: Toxicological assessment and importance of size and composition of particles for oxidative damage and carcinogenic mechanisms. J. Environ. Sci. Health Part C Environ. Carcinog. Ecotoxicol. Rev..

[3] World Health Organization (WHO) (2016). Ambient Air Pollution: A Global Assessment of Exposure and Burden of Disease.

[4] Azarmi F., Kumar P., Mulheron M. (2014). The exposure to coarse, fine and ultrafine particle emissions from concrete mixing, drilling and cutting activities. J. Hazard. Mater..

[5] Xing Y.F., Xu Y.H., Shi M.H., Lian Y.X. (2016). The impact of PM_2.5_ on the human respiratory system. J. Thorac. Dis..

[6] Ghio A.J., Carraway M.S., Madden M.C. (2012). Composition of air pollution particles and oxidative stress in cells, tissues, and living systems. J. Toxicol. Environ. Health Part B Crit. Rev..

[7] Mancebo S.E., Wang S.Q. (2015). Recognizing the impact of ambient air pollution on skin health. J. Eur. Acad. Dermatol. Venereol..

[8] Lensen G., Jungbauer F., Goncalo M., Coenraads P.J. (2007). Airborne irritant contact dermatitis and conjunctivitis after occupational exposure to chlorothalonil in textiles. Contact Dermat..

[9] Strak M., Janssen N.A., Godri K.J., Gosens I., Mudway I.S., Cassee F.R., Lebret E., Kelly F.J., Harrison R.M., Brunekreef B. (2012). Respiratory health effects of airborne particulate matter: The role of particle size, composition, and oxidative potential-the raptes project. Environ. Health Perspect..

[10] Zemp E., Elsasser S., Schindler C., Kunzli N., Perruchoud A.P., Domenighetti G., Medici T., Ackermann-Liebrich U., Leuenberger P., Monn C. (1999). Long-term ambient air pollution and respiratory symptoms in adults (sapaldia study). The sapaldia team. Am. J. Respir. Crit. Care Med..

[11] Ferreira T.M., Forti M.C., de Freitas C.U., Nascimento F.P., Junger W.L., Gouveia N. (2016). Effects of particulate matter and its chemical constituents on elderly hospital admissions due to circulatory and respiratory diseases. Int. J. Environ. Res. Public Health.

[12] Chang C.C., Kuo C.C., Liou S.H., Yang C.Y. (2013). Fine particulate air pollution and hospital admissions for myocardial infarction in a subtropical city: Taipei, Taiwan. J. Toxicol. Environ. Health Part A.

[13] Yeatts K., Svendsen E., Creason J., Alexis N., Herbst M., Scott J., Kupper L., Williams R., Neas L., Cascio W. (2007). Coarse particulate matter (PM_2.5–10_) affects heart rate variability, blood lipids, and circulating eosinophils in adults with asthma. Environ. Health Perspect..

[14] Bandyopadhyay A. (2016). Neurological disorders from ambient (urban) air pollution emphasizing ufpm and PM_2.5_. Curr. Pollut. Rep..

[15] Genc S., Zadeoglulari Z., Fuss S.H., Genc K. (2012). The adverse effects of air pollution on the nervous system. J. Toxicol..

[16] Heusinkveld H.J., Wahle T., Campbell A., Westerink R.H., Tran L., Johnston H., Stone V., Cassee F.R., Schins R.P. (2016). Neurodegenerative and neurological disorders by small inhaled particles. Neurotoxicology.

[17] Maher B.A., Ahmed I.A. (2016). Magnetite pollution nanoparticles in the human brain. Proc. Natl. Acad. Sci. USA.

[18] Sagai M., Win-Shwe T.T. (2015). Oxidative stress derived from airborne fine and ultrafine particles and the effects on brain-nervous system: Part 1. Nihon eiseigaku zasshi. Jpn. J. Hyg..

[19] Vacher G., Niculita-Hirzel H., Roger T. (2015). Immune responses to airborne fungi and non-invasive airway diseases. Semin. Immunopathol..

[20] Holian A., Hamilton R.F., Morandi M.T., Brown S.D., Li L. (1998). Urban particle-induced apoptosis and phenotype shifts in human alveolar macrophages. Environ. Health Perspect..

[21] Balti E.V., Echouffo-Tcheugui J.B., Yako Y.Y., Kengne A.P. (2014). Air pollution and risk of type 2 diabetes mellitus: A systematic review and meta-analysis. Diabet. Res. Clin. Pract..

[22] Brocato J., Sun H., Shamy M., Kluz T., Alghamdi M.A., Khoder M.I., Chen L.C., Costa M. (2014). Particulate matter from saudi arabia induces genes involved in inflammation, metabolic syndrome and atherosclerosis. J. Toxicol. Environ. Health Part A.

[23] Raaschou-Nielsen O., Beelen R., Wang M., Hoek G., Andersen Z.J., Hoffmann B., Stafoggia M., Samoli E., Weinmayr G., Dimakopoulou K. (2016). Particulate matter air pollution components and risk for lung cancer. Environ. Int..

[24] Uccelli R., Mastrantonio M., Altavista P., Caiaffa E., Cattani G., Belli S., Comba P. (2017). Female lung cancer mortality and long-term exposure to particulate matter in Italy. Eur. J. Public Health.

[25] Sun H., Shamy M., Kluz T., Munoz A.B., Zhong M., Laulicht F., Alghamdi M.A., Khoder M.I., Chen L.C., Costa M. (2012). Gene expression profiling and pathway analysis of human bronchial epithelial cells exposed to airborne particulate matter collected from Saudi Arabia. Toxicol. Appl. Pharmacol..

[26] Hougaard K.S., Campagnolo L., Chavatte-Palmer P., Tarrade A., Rousseau-Ralliard D., Valentino S., Park M.V., de Jong W.H., Wolterink G., Piersma A.H. (2015). A perspective on the developmental toxicity of inhaled nanoparticles. Reprod. Toxicol..

[27] Tosh D., Slack J.M.W. (2002). How cells change their phenotype. Nat. Rev. Mol. Cell. Biol..

[28] Maciejczyk P., Chen L.C. (2005). Effects of subchronic exposures to concentrated ambient particles (caps) in mice. VIII. Source-related daily variations in in vitro responses to caps. Inhal. Toxicol..

[29] Duvall R.M., Norris G.A., Dailey L.A., Burke J.M., McGee J.K., Gilmour M.I., Gordon T., Devlin R.B. (2008). Source apportionment of particulate matter in the U.S. and associations with lung inflammatory markers. Inhal. Toxicol..

[30] Kelloff G.J., Lippman S.M., Dannenberg A.J., Sigman C.C., Pearce H.L., Reid B.J., Szabo E., Jordan V.C., Spitz M.R., Mills G.B. (2006). Progress in chemoprevention drug development: The promise of molecular biomarkers for prevention of intraepithelial neoplasia and cancer—A plan to move forward. Clin. Cancer Res..

[31] Kanaji A., Caicedo M.S., Virdi A.S., Sumner D.R., Hallab N.J., Sena K. (2009). Co-cr-mo alloy particles induce tumor necrosis factor alpha production in mlo-y4 osteocytes: A role for osteocytes in particle-induced inflammation. Bone.

[32] Buggiano V., Petrillo E., Alló M., Lafaille C., Redal M.A., Alghamdi M.A., Khoder M.I., Shamy M., Muñoz M.J., Kornblihtt A.R. (2015). Effects of airborne particulate matter on alternative pre-mrna splicing in colon cancer cells. Environ. Res..

[33] Sturn A., Quackenbush J., Trajanoski Z. (2002). Genesis: Cluster analysis of microarray data. Bioinformatics.

[34] Zytoon M.A., Aburas H.M., Abdulsalam M.I. (2014). Determination of 40k, 232th and 238u activity concentrations in ambient PM_2.5_ aerosols and the associated inhalation effective dose to the public in Jeddah city, Saudi Arabia. J. Environ. Radioact..

[35] Balkhyour M.A., Goknil M.K. (2010). Total fume and metal concentrations during welding in selected factories in Jeddah, Saudi Arabia. Int. J. Environ. Res. Public Health.

[36] Elassouli S.M., Alqahtani M.H., Milaat W. (2007). Genotoxicity of air borne particulates assessed by comet and the salmonella mutagenicity test in Jeddah, Saudi Arabia. Int. J. Environ. Res. Public Health.

[37] Uccelli A., Moretta L., Pistoia V. (2006). Immunoregulatory function of mesenchymal stem cells. Eur. J. Immunol..

[38] Denburg J.A., Inman M.D., Wood L., Ellis R., Sehmi R., Dahlback M., O′Byrne P. (1997). Bone marrow progenitors in allergic airways diseases: Studies in canine and human models. Int. Arch. Allergy Immunol..

[39] Denburg J.A., Inman M.D., Sehmi R., Uno M., O′Byrne P.M. (1998). Hemopoietic mechanisms in allergic airway inflammation. Int. Arch. Allergy Immunol..

[40] Oh K.W., Moon C., Kim H.Y., Oh S.I., Park J., Lee J.H., Chang I.Y., Kim K.S., Kim S.H. (2015). Phase I trial of repeated intrathecal autologous bone marrow-derived mesenchymal stromal cells in amyotrophic lateral sclerosis. Stem Cells Transl. Med..

[41] Gao F., Chiu S.M., Motan D.A., Zhang Z., Chen L., Ji H.L., Tse H.F., Fu Q.L., Lian Q. (2016). Mesenchymal stem cells and immunomodulation: Current status and future prospects. Cell Death Dis..

[42] Jiang M., Stanke J., Lahti J.M. (2011). The connections between neural crest development and neuroblastoma. Curr. Top. Dev. Biol..

[43] Howlader N., Krapcho M., Garshell J., Neyman N., Altekruse S.F., Kosary C.L., Yu M., Ruhl J., Tatalovich Z., Cho H. (2012). Seer Cancer Statistics Review, 1975–2010; SEER Web Site.

[44] Humphries F., Yang S., Wang B., Moynagh P.N. (2015). Rip kinases: Key decision makers in cell death and innate immunity. Cell Death Diff..

[45] Hunter P. (2012). The inflammation theory of disease. The growing realization that chronic inflammation is crucial in many diseases opens new avenues for treatment. EMBO Rep..

[46] Cui Y., Xie X., Jia F., He J., Li Z., Fu M., Hao H., Liu Y., Liu J.Z., Cowan P.J. (2015). Ambient fine particulate matter induces apoptosis of endothelial progenitor cells through reactive oxygen species formation. Cell. Physiol. Biochem..

[47] Monn C., Becker S. (1999). Cytotoxicity and induction of proinflammatory cytokines from human monocytes exposed to fine (PM_2.5_) and coarse particles (PM_10–2.5_) in outdoor and indoor air. Toxicol. Appl. Pharmacol..

[48] Tan H.H., Fiel M.I., Sun Q., Guo J., Gordon R.E., Chen L.C., Friedman S.L., Odin J.A., Allina J. (2009). Kupffer cell activation by ambient air particulate matter exposure may exacerbate non-alcoholic fatty liver disease. J. Immunotoxicol..

[49] Michael S., Montag M., Dott W. (2013). Pro-inflammatory effects and oxidative stress in lung macrophages and epithelial cells induced by ambient particulate matter. Environ. Pollut. (Barking Essex: 1987).

[50] Yi S., Zhang F., Qu F., Ding W. (2014). Water-insoluble fraction of airborne particulate matter (PM_10_) induces oxidative stress in human lung epithelial a549 cells. Environ. Toxicol..

[51] Xiao G.G., Wang M., Li N., Loo J.A., Nel A.E. (2003). Use of proteomics to demonstrate a hierarchical oxidative stress response to diesel exhaust particle chemicals in a macrophage cell line. J. Biol. Chem..

[52] Teeguarden J.G., Hinderliter P.M., Orr G., Thrall B.D., Pounds J.G. (2007). Particokinetics in vitro: Dosimetry considerations for in vitro nanoparticle toxicity assessments. Toxicol. Sci..

